# Risk factors for mediastinitis after cardiac surgery – a retrospective analysis of 1700 patients

**DOI:** 10.1186/1749-8090-2-23

**Published:** 2007-05-20

**Authors:** Claudius Diez, Daniel Koch, Oliver Kuss, Rolf-Edgar Silber, Ivar Friedrich, Jochen Boergermann

**Affiliations:** 1 Department of Cardiothoracic Surgery, Martin-Luther-Universität Halle-Wittenberg, Ernst-Grube-Str. 40, D-06097 Halle/Saale, Germany; 2 Institute of Medical Epidemiology, Biometrics and Informatics, Martin-Luther-Universität Halle-Wittenberg, Magdeburger Str. 27, D-06097 Halle/Saale, Germany

## Abstract

**Background:**

Mediastinitis is a rare, but serious complication of cardiac surgery. It has a significant socioeconomic impact and high morbidity. The purpose of this study was to determine pre-, intra-, and postoperative predictors of mediastinitis.

**Methods and results:**

In 1700 consecutive patients, who underwent cardiac surgery in 2001, 49 variables were retrospectively assessed. Forty-five patients (2.65%, 95% CI [1.88; 3.41]) developed postoperative mediastinitis. None of these patients died during their hospitalization. Multivariate analysis identified three of the 49 variables as highly significant independent predictors for the development of mediastinitis: obesity (OR 1.03, 95% CI [1.01; 1.04] p = 0.001), chronic obstructive pulmonary disease (OR 3.30, 95% CI [1.58; 6.88], p = 0.001), and bilateral grafting of the internal mammary artery (OR 3.18, 95% CI [1.20; 8.43] p = 0.02). The model is reliable in terms of its goodness of fit, it also discriminates well. Additionally, univariate analysis identified diabetes mellitus, CCS class and the number of intraoperatively transfused units of fresh frozen plasma as variables with a significant impact.

**Conclusion:**

The present study suggests that bilateral IMA grafting, chronic obstructive pulmonary disease and obesity are important predictors of mediastinitis.

## Background

As a severe complication of cardiac surgery, mediastinitis continues to be associated with tremendous morbidity and cost [1]. The exact pathogenetic mechanisms underlying postoperative mediastinitis remain unknown; multiple factors might play a role. Some authors favor the intraoperative contamination [2], whereas other studies demonstrated that endogenous bacteria might be pathogenetically involved [3,4] because preoperative intranasal antibiotic treatment significantly reduced the incidence of mediastinitis.

Several studies published during the last ten years reported an incidence of 0.4 to 5% [5,6] and in-hospital mortalities between 14 and 23%, even when mediastinitis was correctly treated [7-9]. Postoperative mediastinitis is also associated with high long-term mortality [10,11]. Braxton et al. compared in a 4-year follow-up study the survival rates of patients with and without mediastinitis after cardiac operations. Eighty nine percent of patients survived in the non-mediastinitis group compared to 65% in the mediastinitis group after four years [10].

In 1963 Shumacker and Mandelbaum first described a method for the treatment of postoperative mediastinitis [12] which still forms the basis for current therapeutic approaches in some centers [13]. Their approach included early surgical debridement, insertion of a drainage system with continuous irrigation with antibiotic solution and primary wound closure.

Modern management of mediastinitis with early aggressive debridement followed by delayed wound closure has been reported to reduce early mortality to less than 20%.

The vacuum-assisted closure (VAC) was introduced in 1997 [14] and combines the advantages of both open and closed treatment. Besides an improved local perfusion and oxygenation, the quantitative bacterial flora is rapidly reduced and the formation of scar tissue is stimulated [15,16]. Intermittent suction additionally may promote wound healing by local reactive hyperemia if suction stops [17]. Several studies showed the benefits of a VAC therapy in terms of shorter hospitalizations, earlier secondary wound closure and a lower mortality compared to a conventional therapy [18-20]. However, evidence-based guidelines for the treatment of postoperative mediastinitis have not been established and published yet.

In this study, we evaluated pre-, intra-, und postoperative risk factors for mediastinitis and compared the results with a previous report from our department [21]. This report analysed 112 mediastinitis patients from 1988 to 1999. There were two major reasons for a new mediastinitis study from our department. First, we wanted to know how several surgical modifications at the end of 1990s in our clinic (e.g. more frequent IMA use, sternal closure with wires instead of sutures) influenced the prevalence of postoperative mediastinitis. Second, we wanted to examine if the increased incidence of multiple comorbidities among our patients during the recent decade (e.g. increased age, more obese patients) may lead to an increase in mediastinitis after cardiac surgery.

## Material, methods and statistics

### Patients

The study group consisted of 1700 patients who underwent cardiac surgery with or without extracorporeal circulation. Patients were recruited from the Department of Cardiothoracic Surgery at the University of Halle-Wittenberg and an associated private heartcenter. Patients undergoing sole CABG, sole valve replacement/repair, or combined CABG/valve procedures were included in the analysis.

The surgical procedures among our study population were as follows: sole CABG (n = 1438), sole valve procedure (n = 155), combination of CABG and valve procedure (n = 89) and other procedures, e.g. ASD closure, (n = 18). IMA harvesting was performed with a pedicle in 85% (n = 1029) and 15% in a skeletonized way (n = 182). The prophylactic antibiotic regimen in both centers included the intravenous administration of 2 g Cefotiam one hour preoperatively and six hours postoperatively. Cefotiam was usually continued in all patients with valve procedures (2 g iv tid) until all chest tubes and the central venous catheter were removed.

### Data collection and definition of variables

Data were retrospectively collected from patient records and entered into an Excel spreadsheet. For most variables, data were missing in fewer than 0.5% of patients.

Where appropriate, variables such as duration of cardiopulmonary bypass and duration of artificial ventilation were analyzed as continuous parameters. All other variables were analyzed as categorical variables. All patients in the mediastinitis group had purulent deep sternal wound infections requiring extensive debridement and drainage as well as bacteriological analyses. Superficial wound infections sparing the sternum and not requiring extensive debridement and drainage were not classified as mediastinitis. Microbiological assessment of intraoperative wound swabs was performed at the Institute of Medical Microbiology of the University of Halle or in the Laboratory Dr. Reising-Ackermann in Leipzig, Germany.

Where appropriate, we used the EuroSCORE-criteria to define the variables; e.g. renal failure was considered to be present when serum creatinine levels exceeded 200 μmol/l. The term "diabetes" was used for all patients with diabetes, insulin dependent and non-dependent. Chronic obstructive pulmonary disease refers to disease with long-term bronchodilator or steroid therapy.

### Mediastinitis treatment

Patients with suspected and/or manifest mediastinitis underwent aggressive surgical exploration with debridement of infected soft tissue and bone using a rongeur and removal of all sternal wires. Two sets of wound swabbings were sent for culture. The wound was then irrigated with antibiotic solution. Nine patients received initial rewiring of the sternum by the method described by Robiscek [22] and insertion of a drainage system followed by bilateral pectoralis muscle mobilization to cover the sternum. Subcutaneous tissue and skin were closed with single sutures. The wound was irrigated continuously with antiseptic solution (0,5% povidone-iodine; 4 liters per 24 hours) for at least three days followed by a continuous saline wash (4 liters per 24 hours) for 72 hours. Swabbings from the saline wash were taken every 24 hours and if they were sterile, the catheters were removed stepwise. None of the patients underwent a reoperation.

However, the majority of patients received a vacuum-assisted closure (100 mm Hg suction) after aggressive debridement to expedite the healing process. The foams were changed every third day with concomitant wound swabbings. After sufficient healing, the wounds were either closed with sternal rewiring and muscle flap reconstruction (n = 30) or, in case of loss of sternal bone, with an omentum plastic (n = 6) and secondary wound healing.

The results from the wound swabbings dictated the antibiotic regimen. Patients with suspected and/or manifest mediastinitis initially received Piperacilline 2 g iv tid in combination with a β-lactamase inhibitor and, if necessary, the regimen was changed according to the resistogrammes.

### Statistics

Statistical evaluation was performed using SPSS 11 for Windows [23]. After testing for (non)-normality with the Shapiro-Wilk test, continuous parameters were analyzed with non-parametric tests for independent groups. Categorical variables were analyzed with the Chi-Square-test or, where appropriate, with Fisher's exact test. A p-value < 0.05 was considered significant. Predictors of mediastinitis were analyzed with multivariate logistic regression (stepwise forward algorithm); goodness of fit was tested with the Hosmer-Lemeshow test. A Receiver Operating Characteristic analysis (ROC) with the data from the logistic regression was used to determine if the model sufficiently discriminates between patients with and without mediastinitis.

Data are shown either as mean ± standard deviation or median with range.

## Results

### Incidence, hospital stay and in-hospital mortality

Forty-five of 1700 patients operated on in 2001 (2.65%) developed postoperative mediastinitis. Twelve patients (27%) had already been transferred to the rehabilitation facilities and were readmitted for the treatment of postoperative mediastinitis. The mean postoperative length of stay was 43 ± 35 days (95% CI: 32; 54) for the mediastinitis group compared to 19 ± 17 days (95% CI: 18; 20) for patients without mediastinitis (p < 0.001). None of the 45 patients in the mediastinitis group died, whereas 73 patients in the non-mediastinitis group (4.4%) expired during their hospital stay.

### Preoperative risk factors analysis

By univariate analysis, the following six variables were associated with an increased risk of mediastinitis (p < 0.05): body mass (respectively body mass index), chronic obstructive pulmonary disease (COPD), CCS class, bilateral IMA use and diabetes mellitus (Table 1).

**Table 1:** Prevalence of preoperative risk factors

Variable	Mediastinitis (n)		Σ (n)	p
			
	No	Yes		
Age (years)	64.1 ± 10.2	66.5 ± 8.73	1700	0.71
Gender				
Male	1175	37	1212	n.s.
Female	479	8	487	
Body mass index (kg/m^2^)	28.0	29.8	1700	0.008
Hypertension				
No	273	7	280	n.s.
Yes	1381	38	1419	
Diabetes mellitus				
No	1086	23	1109	0.05
Yes	568	22	590	
Active Smoking				
No	858	15	873	n.s.
Yes	295	11	306	
COPD				
No	1507	35	1542	0.006
Yes	147	10	157	
Peripheral vascular disease				
No	1465	39	1504	n.s.
Yes, untreated	132	3	135	
Yes, treated	56	3	59	
Ejection fraction (% ± SD)	59.6 ± 15.8	58.7 ± 15.0	1700	n.s.
Previous cardiac surgery				
No	1607	45	1652	n.s.
Yes	47	0	47	
NYHA class				
I	113	3	116	n.s.
II	882	19	901	
III	566	20	586	
IV	55	2	57	
CCS class				
I	100	0	100	0.02
II	725	15	740	
III	686	23	709	
IV	104	7	111	
Renal insufficiency				
No	1318	30	1348	n.s.
Yes, compensated	309	14	323	
Yes, dialysis	16	1	17	
Status post renal transplantation	4	0	4	
Hemoglobin (mmol/l ± SD)	8.6 ± 1.1	8.4 ± 1.2	1697	n.s.
Creatinine (μmol/l ± SD)	96.7 ± 64.9	109.1 ± 58.7	1690	n.s.
Blood urea nitrogen (mmol/l ± SD)	6.8 ± 3.2	7.5 ± 4.4	1672	n.s.
Surgical technique				
cardiopulmonary bypass	1276	32	1308	n.s.
OPCAB	276	9	285	
PACAB	18	0	18	
MIDCAB	84	4	88	
Number of bypasses (n ± SD)	2.6 ± 1.2	2.7 ± 1.1	1699	n.s.
Use of LIMA				
No	479	9	488	n.s.
Yes	1175	36	1211	
Bilateral IMA use				
No	1587	40		0.02
Yes	67	5		
Total arterial revascularization				
No	1276	31	1307	n.s.
Yes	378	14	392	

Prevalence of preoperative risk factor among the study population. Data are shown either as absolute numbers or as mean ± SD. Besides several demographic data, there are also variables describing clinical conditions and surgical technique.

### Perioperative risk factor analysis

We examined the following perioperative parameters: urgency (elective, urgent, emergency), use of intraaortic balloon counterpulsation, operative time, duration of cardiopulmonary bypass, catecholamines dose, transfusion of red cell or platelet concentrates, number of transfused units of fresh frozen plasma, and lowest intraoperative body temperature. Only the number of intraoperatively transfused units of fresh frozen plasma proved to be a statistically significant risk factor for the development of mediastinitis (598 ± 361 ml versus 1600 ± 0 ml). This finding is based on one single patient, however.

### Postoperative risk factor analysis

Postoperative variables included postoperative bleeding, body temperature, catecholamine dose, cardiac enzyme levels (creatinine kinase, troponin I), and duration of artificial ventilation. None of these variables showed a significant difference between the groups with versus without mediastinitis.

### Multivariate logistic regression model

The significant variables except FFP transfusion and some almost significant variables (p < 0.1) from the univariate analysis (peripheral arterial disease, preoperative hemoglobin level, duration of operation, duration of bypass, postoperative intraaortic balloon counterpulsation) were included in the multivariate logistic regression analysis. Three independent predictors of mediastinitis were identified (Table 2).

**Table 2:** Final multivariate model

Variable	B	SD	Wald	P (Wald)	Odds Ratio	95% CI	
Body mass index	0.03	0.009	10.51	0.001	1.03	1.012	1.049
COPD	1.19	0.37	10.17	0.001	3.30	1.58	6.88
BIMA use	1.15	0.49	5.44	0.020	3.18	1.20	8.43
Constant	-6.33	0.82	59.17	< 0.0001	0.002		

Final multivariate model. B denotes the coefficient derived from computed stepwise logistic regression and the Odds ratio is statistically e^coefficient ^and describes the probability of having a mediastinitis in the presence of a risk factor. The Wald test was obtained by comparing the maximum likelihood estimate of the slope parameter to an estimate of its standard error. SD is the standard error of B and the 95% CI refers to the Odds ratio.

**Figure 1:**
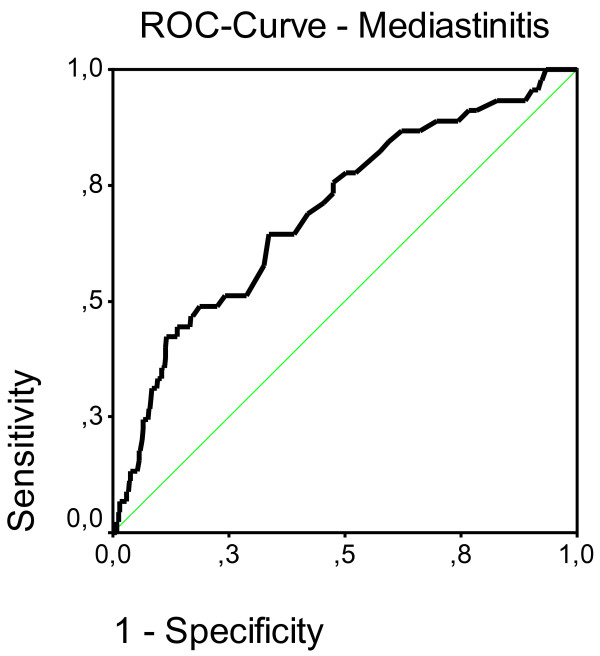
ROC-Diagram of sensitivity versus 1-specificity for all possible cut-off values. The area under the curve (AUC) is 69.3% [95% CI 61.3, 77.4], p < 0.0001), which translates into acceptable discrimination of the final model.

Goodness of fit was tested with the Hosmer-Lemeshow test (third step: p = 0.59, χ^2 ^= 6.47). The generated model showed a useful goodness of fit. To discriminate between patients with and without mediastinitis, a ROC analysis was performed with the data from the logistic regression (Figure 1).

### Bacteriology

S. aureus was isolated from 16 wounds (32%) and S. epidermidis from 12 wounds (24%). From the remaining wounds, several other bacterial species could be isolated, including E. coli, Klebsiella spp., Pseudomonas aeruginosa, Enterococcus faecalis, Serratia marcescens, and Corynebacterium species. In one mediastinitis patient, no bacteria could be initially isolated, but S. aureus was found in a swabbing obtained three days later during a foam exchange for VAC therapy. In six patients, we identified a mixed mediastinal flora.

## Discussion

Since it was first introduced by Julien et al. in 1957, median sternotomy has been established as the standard approach for open heart surgery [24]. Mediastinitis continues to be a major complication of cardiac surgery and is associated with tremendous morbidity and cost. The incidence of postoperative mediastinitis varied between 0.5 and 5% in several studies. Our reported incidence of 2.65% is in agreement with recently published results [21,25]. Long-term mortality has been extensively investigated in several studies. Ridderstolpe et al. showed a 30-day mortality of 1%, and a mortality of 7.2% after one year of follow-up.

The economic impact of postoperative mediastinitis has also been evaluated in several studies, most of which were conducted in the United States. Very few studies apply to German hospitals. Our data show a significantly prolonged hospital stay. On the average, patients with mediastinitis were hospitalized 24 days longer than patients without this complication. Hollenbeak et al. reported that the mediastinitis treatment required 20 additional hospital days [1]. Our data come quite close to the data from this study.

During the recent ten years, several studies evaluated risk factors for postoperative mediastinitis [2,7-9,21,25-40]. It is of note, however, that low patient numbers and multiple statistical tests can lead to false positive results [41]. While the above mentioned studies did identify numerous predisposing factors, they also led to some contradictions, primarily because mediastinitis, deep sternal wound infection, and sternal instability were defined differently from one study to the next. This is compounded by geographical differences, different study durations and varying endpoints.

The most frequently mentioned risk factor is obesity. Our data also show that the risk for mediastinitis increases by three percent per additional kilogram body mass per square meter body surface. Why obesity presents a risk factor was discussed by Milano et al. and Bitkover et al. [2,41]. The etiology relates to increased postoperative mechanical loads, facilitated bacterial contamination, and failure to adjust antibiotic doses to body mass, an error that leads to inappropriately low tissue antibiotic concentrations. Additionally, the bradytrophic properties of fatty tissue contribute to poor wound healing.

Our data also link bilateral IMA use to an increased risk of postoperative mediastinitis. This is explained by reduced sternal perfusion after IMA dissection [34]. One animal study demonstrated that sternal blood flow was decreased by 90% after bilateral IMA dissection [42]. Carrier et al. showed that after bilateral IMA dissection, sternal perfusion was reduced by 24 ± 6%. Four weeks postoperatively, sternal blood flow was still reduced by 2 ± 2% [43]. Low sternal perfusion may lead to tissue necrosis and can impair wound healing. This is contradicted by several studies which demonstrated that bilateral IMA use is not associated with increased rates of postoperative mediastinitis [27,44]. Both authors skeletonize the IMA instead of dissecting it with a pedicle. Even diabetic patients benefit from skeletonized IMA dissection [45,46]. IMA harvesting might indeed contribute to the development of postoperative mediastinitis in our study because the majority of this conduit was dissected as a pedicle.

The third independent predisposing factor in our study population was COPD, which was associated with a more than threefold increased risk of postoperative mediastinitis, possibly as a result of frequent coughing, which might contribute to wound dehiscence and thereby facilitate bacterial migration [38]. Patients with COPD also experience more frequent respiratory infections and prolonged weaning from artificial ventilation [33,40,47].

We also compared the identified risk factors with data from a recent report from our department [21]. The only risk factor common to both studies was obesity, whereas low cardiac output, smoking, age, postoperative respiratory failure and the type of sternal closure were not related to the risk of postoperative mediastinitis.

It is noteworthy that we found no difference in the prevalence of mediastinitis between operations with or without extracorporeal circulation (Off-pump).

Our data also demonstrate the need for preventive measures. Weight reduction (in case of elective surgery), weight adapted perioperative antibiotic treatment and a strict antidiabetic regimen proved to be effective procedures [17,22,24].

## Conclusion

We conclude from our study that bilateral IMA grafting, chronic obstructive pulmonary disease with long-term bronchodilator therapy and obesity are important predictors of mediastinitis after cardiac surgery.

## Abbreviations

ASD – atrial septal defect, COPD – chronic obstructive pulmonary disease, (L)IMA – (left) internal mammary artery, OR – Odds ratio, ROC – Receiver operating characteristics, VAC – Vacuum assisted closure.

## Competing interests

The author(s) declare that they have no competing interests.

## Authors' contributions

CD, IF and JB designed the study and developed the database. CD transferred the data into SPSS, performed the statistical analysis and drafted the manuscript.

JB critically revised the manuscript in cooperation with the co-authors and interpreted the data. CD and JB contributed equally to this study.

DK examined the patient files and collected the data in an Excel-sheet. He also was involved in the coordination and interpretation of the data.

OK considerably helped with the advanced statistical analysis (multivariate analysis and ROC) and the interpretation of data.

IF revised the manuscript and analysed the statistical data.

RES as the department chair supported this study, participated in designing the study and critically revised the manuscript.

All authors have read and approved the submitted version of the manuscript.
